# Genome-wide analysis of mutations induced by carbon ion beam irradiation in cotton

**DOI:** 10.3389/fpls.2023.1056662

**Published:** 2023-02-16

**Authors:** Jianguang Liu, Guiyuan Zhao, Jinpeng Geng, Zhao Geng, Haikuan Dou, Xu Liu, Zetong An, Hanshuang Zhang, Yongqiang Wang

**Affiliations:** ^1^ Institute of Cotton, Hebei Academy of Agriculture and Forestry Sciences/Key Laboratory of Biology and Genetic Improvement of Cotton in Huanghuaihai Semiarid Area, Shijiazhuang, China; ^2^ School of Science, Hebei University of Technology, Tianjin, China

**Keywords:** cotton, carbon-ion beam irradiation, genetic variation, mutants, whole genome re-sequencing

## Abstract

Carbon ion beam (CIB) irradiation is a powerful way to create mutations in animals, plants, and microbes. Research on the mutagenic effects and molecular mechanisms of radiation is an important and multidisciplinary issue. However, the effect of carbon ion radiation on cotton is uncertain. In this study, five different upland cotton varieties and five CIB doses were used to identify the suitable irradiation dose for cotton. Three mutagenized progeny cotton lines from the wild-type Ji172 were re-sequenced. The effect of half-lethal dose on mutation induction indicated that 200 Gy with LET_max_ of 226.9 KeV/μm was the most effective heavy-ion dose for upland cotton and a total of 2,959-4,049 single-base substitutions (SBSs) and 610-947 insertion-deletion polymorphisms (InDels) were identified among the three mutants by resequencing. The ratio of transition to transversion in the three mutants ranged from 2.16 to 2.24. Among transversion events, G:C>C:G was significantly less common than three other types of mutations (A:T>C:G, A:T>T:A, and G:C>T:A). The proportions of six types of mutations were very similar in each mutant. The distributions of identified SBSs and InDels were similar with unevenly distributed across the genome and chromosomes. Some chromosomes had significantly more SBSs than others, and there were “hotspot” mutation regions at the ends of chromosomes. Overall, our study revealed a profile of cotton mutations caused by CIB irradiation, and these data could provide valuable information for cotton mutation breeding.

## Introduction

Upland cotton is one of the most important commercial crops worldwide, and contributes more than 90% of global cotton production (Grover et al., 2015). However, artificial selection for a few traits in cotton germplasm by traditional breeding methods has resulted in narrow genetic diversity among cotton varieties. Consequently, the relatively narrow genetic base restricts the progress of the cotton breeding (Zhang et al., 2008; Saleem et al., 2020; Santhy et al., 2019; Zhang et al., 2020).

Mutagenesis is a powerful tool for creating new germplasm resources and to clarify the function of plant genes (Holme et al., 2019; Bhoi et al., 2022). Various artificial mutagenic techniques, such as T-DNA insertion mutations and physical mutagens, have been widely used in many kinds of plants. T-DNA insertion mutations are usually applied to model plants such as *Arabidopsis* and rice because these plants have established genetic transformation techniques (Alonso et al., 2003; Lorieux et al., 2012). However, this approach is too impractical, time-consuming, and expensive to apply to cotton because of genetic transformation barriers.

Ethyl methanesulfonate (EMS) and sodium azide (NaN_3_), as the most common chemical mutagens (Luan et al., 2007; Wang et al., 2014; Lu et al., 2016; Chen et al., 2018; Espina et al., 2018), have been applied to cotton (Xu et al., 2011; Witt et al., 2018). These approaches mainly generate point mutations with high frequency throughout the whole genome (Patel et al., 2022). Upland cotton EMS mutant libraries currently include approximately 12,000 plants and more than 20 mutant phenotypes (Lian et al., 2020). To expand the population and increase phenotypic variation, Yang et al. obtained a mutant library of 23,000 M1 plants by EMS mutagenesis (Yang et al., 2019). In addition, a candidate gene (*Gh_D05G364200*) for crumpled leaves was screened by BSA and transcriptome sequencing (Wei et al., 2022).

Radiation-induced mutagenesis is also the most common method for developing direct mutant varieties (Yamaguchi et al., 2003; Kikuchi et al., 2009; Yamaguchi et al., 2010; Ishikawa et al., 2012; Kazama et al., 2013). Carbon ion beam (CIB) irradiation, a typical form of heavy-ion beam irradiation, has higher linear energy transfer (LET), and can deposit more energy at a designated depth; additionally, it has advantages for creating mutants (Permata et al., 2021), such as high mutation efficiency and a broad mutation spectrum. Heavy-ion beam irradiation can generate mutations in the form of substitutions, small insertion–deletion polymorphisms (InDels), and structure variants. Currently, it has been used for mutation breeding or genetic studies in many crops, such as wheat(Zhang et al., 2008; Fitzgerald et al., 2015; Li et al., 2022), rice(Belfield et al., 2012; Yang et al., 2019; Zhang et al., 2021), *Arabidopsis thaliana *(Du et al., 2014; Du et al., 2017; Du et al., 2018), chrysanthemum (Yamaguchi et al., 2010), and soybean (Im et al., 2017; Kim et al., 2008). Many plant variations have been induced by CIB irradiation, and many novel experimental materials and practical cultivars have been generated. The DNA mutation rate of sweet sorghum was reported to be 18.95% at 200 Gy, while the mutation rate decreased to 17.87% at 240 Gy, with effects on phenotype mainly on node number, plant height, stalk diameter, sugar content and single stem weight (Dong et al., 2015). Luo et al. (2016) showed that 400 Gy as the median lethal dose (LD50) could be used for a large-scale mutant screening in Lotus japonicus, 27 morphological mutants were generated including leaf, stem, flower and fruit phenotypic variation (Luo et al., 2016). Soybean seeds treated with carbon ion beam irradiation at a dose of 120 Gy (80 Mev/u) resulted in the most extensive variation and can be effectively used for soybean mutation breeding (Wang et al., 2021). These results suggested that CIB irradiation was an effective method for creating mutants, and that different species varied in their sensitivities to ionic radiation. However, CIB irradiation has not been used for cotton, and the biological effects of CIB irradiation on cotton have not been elucidated.

In this study, we investigated the mutational effects of different doses of heavy-ion radiation on plants and re-sequenced three mutagenized lines from CIB irradiation by next-generation sequencing. The pattern of mutation distribution along the chromosome and the base changes of these gene mutations were further investigated, and provided more information on the mechanism of CIB mutagenesis in upland cotton.

## Materials and methods

### Plant material and CIB irradiation

The dry seeds of the five upland cotton cultivated varieties Ji172, NDM13, 17Xi, Ji178, and LZY10 were placed on a plastic plate in a monolayer with the chalazal pole upward when irradiating and the cotton germ close to the ion source. The seeds were irradiated with ^12^C^6+^ ions at doses of 100 Gy, 150 Gy, 200 Gy, 300 Gy, and 500 Gy. The ray energy was set to 87.5 MeV (LET_max_, 226.9 KeV/μm).

To promote germination, the irradiated seeds were placed on wet filter papers at 28°C for 48 h, and then transferred into pots with nutritional soil and vermiculite (v/v = 1:1), and grown in a greenhouse at 25°C with a 16 h light/8 h dark photoperiod until the cotyledon expanded. Then, the seedlings were transplanted into the cotton field.

To determine the half-lethal dose (LD50), the relative seed germination rate (relative seed germination rate = germination of irradiated seeds/germination of control seeds) was calculated for the five cultivated cotton varieties. M_2_ seeds were collected from the M_1_ plants. The visible phenotypic mutants were selected from 9,073 M_2_ populations throughout the development period. In late April, the selected mutant progeny were sown in a plot to become a line. Those plants with traits not in accordance with the target traits were eliminated, and the remaining plants were then self-pollinated. This process of mutant selection was repeated from the M3 to M8 generation. The stably inherited mutants were saved for the next step in the study.

### Whole-genome re-sequencing and variant detection

Three visible phenotypic mutants were observed: small leaf, yellow leaf, and semi-dwarf mutants (M_8_). These mutants, in addition to wild-type (WT) plants (Ji172), were chosen for whole-genome re-sequencing. Young leaves from the above five samples were collected and the genomic DNA was extracted using the DNeasy Plant Mini Kit (Qiagen, Hilden, Germany). The cotton genomic re-sequencing was carried out on an Illumina HiSeq™ 2500 system (Illumina, Inc., San Diego, CA, USA) at Biomics Technologies Company (Beijing, China). The experimental procedures were performed according to the standard protocols provided by Illumina for sample quality testing, library construction, library quality testing, and library sequencing. The raw data were filtered through quality assessment by the standard Illumina process. Then, the obtained clean data were mapped to the upland cotton reference genome of TM-1 (https://www.cottongen.org/species/Gossypium_hirsutum/nbi-AD1 _genome_v1.1) with the BWA-MEM (version 0.7.16a) algorithm (Li and Durbin, 2009). We applied the Genome Analysis Toolkit (GATK, version 4.0.4.0) procedures to call single-base substitutions (SBSs) and InDels. After removing the duplicate reads with Picard MarkDuplicates, we called the SBSs and InDels using HaplotypeCaller. To obtain high-quality SBSs and InDels from each sample, the variant calls from GATK were subsequently filtered with the parameters QD < 2.0, FS > 60.0, MQ < 20.0, MQRankSum < −12.5, and ReadPosRankSum < −8.0; then, the variants were filtered by vcftools with the parameters max-missing 1.0 and minDP 8. Variants with an allele frequency of 25%–75% were considered heterozygous, and those with an allele frequency > 75% were considered homozygous. Only those mutation sites that differed from the WT and other mutants were considered reliable mutation sites.

### Verification of the mutation sites

To verify whether the mutation sites were indeed correct, Sanger sequencing was employed to estimate the accuracy of our mutation calls. The specific primers were designed around the mutations using Primer5 to amplify a 400-800 bp region (**Table S1**). Three mutant as well as WT genomic DNA were extracted using the DNeasy Plant Mini Kit (Qiagen, Hilden, Germany). PCR amplification was performed with an initial denaturation step at 95°C for 5 min, followed by 35 cycles at 94°C for 30s, 52-56°C for 30s, and 72°C for 30s, with a final extension step at 72°C for 10 min. Only the aimed specific PCR products were used for Sanger sequencing. The sequence alignment were performed by DNAMAN 6.0.3.48.

## Results

### Suitable dose of CIB irradiation and abundance of phenotypic mutants

The LD50 is generally considered a key parameter for mutagenesis. In this study, dry seeds from five upland cotton varieties were irradiated with five doses of CIB ranging from 100 Gy to 500 Gy (**Table 1**). The relative seed germination rate of these genotypes were recorded to determine the LD50. The results showed that seed germination rates for all five varieties linearly decreased with increasing CIB dose. The germination rate ranged from 71.3% to 95.2% in 100 Gy progenies, 55.8% to 74.3% in 150 Gy progenies, 48.3% to 61.6% in 200 Gy progenies, 31.2% to 46.4% in 300 Gy progenies, and 0.0% to 29.4% in 500 Gy progenies (**Table 1**). Moreover, under the same radiation dose, different changes in germination rate were observed for the five varieties. At a dose of 100 Gy, the relative germination rate of Ji178 was as high as 95.2%, whereas that of NDM13 was only 71.3%. At a dose of 500 Gy, the relative germination rate of JI178 was 15.9%, but no seeds germinated in the progeny of irradiated NDM13 (**Table S2**). Based on germination rate, the LD50s of JI172, 17Xi, Ji178, and LZY10 were between 200 Gy and 300 Gy, whereas the LD50 of NDM13 was between 150 Gy and 200 Gy. In addition, we found no phenotypic mutants at 100 Gy and the greatest number of phenotypic mutants was found at 200 Gy (**Table S2**). However, as the radiation dose continued to increase, the number of phenotypic mutants decreased, and only one sterile mutant and one lethal mutant were found at 500 Gy. Therefore, 200 Gy with 226.9 KeV/μm LET_max_ is recommended as the optimum CIB radiation dose for cotton mutagenesis.

**Table 1:** The relative surviving plant rate at different doses.

Irradiated dose	100Gy	150Gy	200Gy	300Gy	500Gy
Cotton varieties					
Ji172	79.8%	70.3%	58.3%	46.4%	24.3%
NDM13	71.3%	55.8%	48.3%	42.3%	0.0%
17Xi	84.6%	74.3%	53.3%	38.3%	29.4%
Ji178	95.2%	71.2%	61.6%	31.2%	15.9%
LZY10	84.2%	70.2%	59.0%	31.6%	5.3%

**Figure 1:**
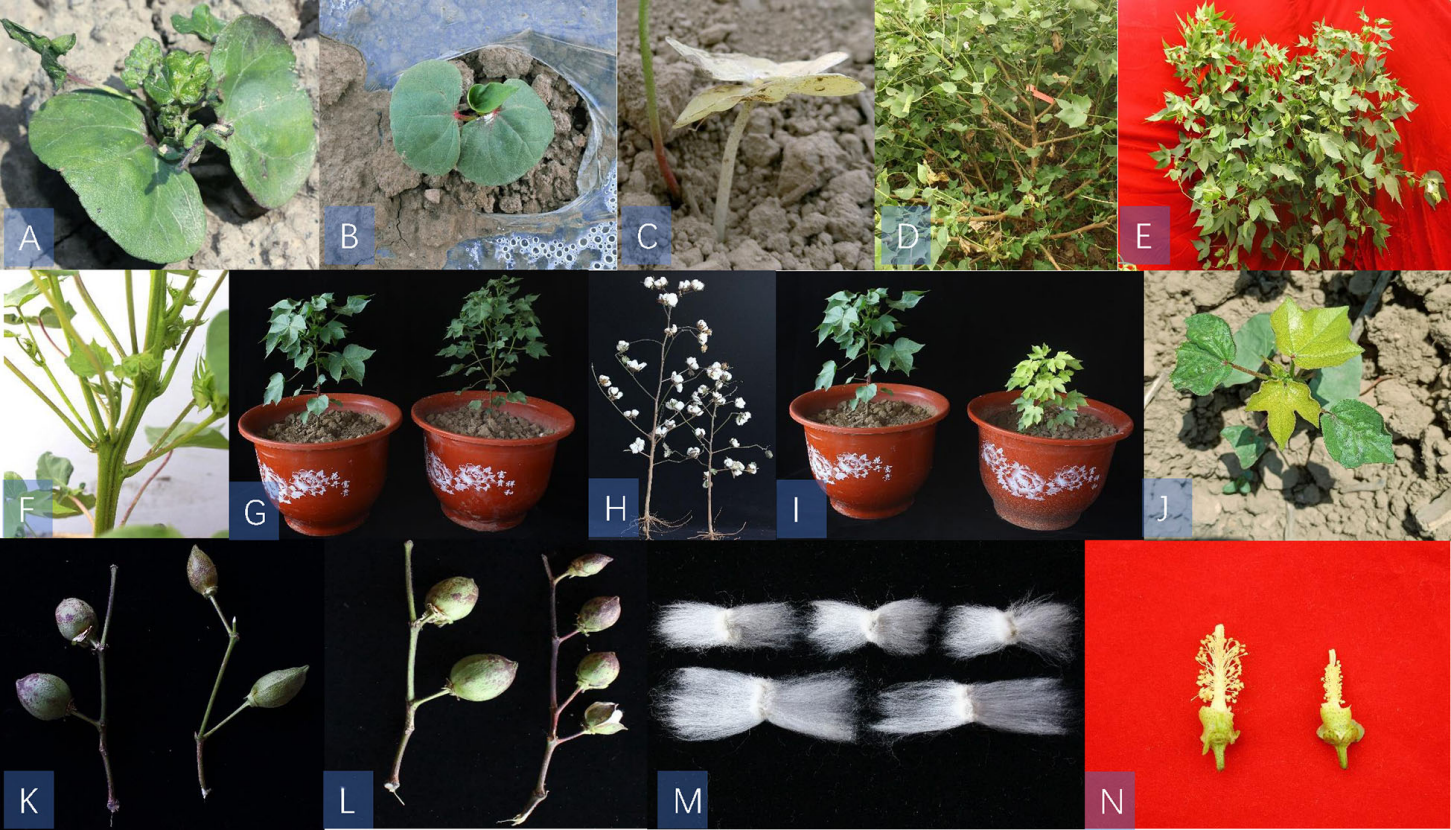
Phenotypic mutants in M2 populations. Panels **(A-C)**: lethal mutants; panels **(D, E)**:sterility mutants; panels **(F-H)**: plant architecture mutants; panels **(I, J)**: leaf color mutants, panels **(K, L)**: boll stalk and boll shape mutants, **(M);** fibre mutant and **(N);** flower style mutant. panels **(G, H, I, K, L, N)**: WT is on the left and mutants is on the right; **(M);** mutants is below and WT is above.

We also identified several phenotypic mutants in the M_2_ populations, such as lethal (**Figures 1A–C**), sterile (**Figures 1D, E**), plant architecture (**Figures 1F–H**), leaf color (**Figures 1I, J**), boll stalk and boll shape (**Figures 1K, L**), fiber mutants (**Figure 1M**), and flower mutants (**Figure 1N**). In the plant architecture mutants, there were relatively more bolls and smaller leaves compared with WT plants (**Figure 1G**). We also observed a semi-dwarf mutant that had shorter internode lengths and shorter fruit branches compared with WT plants (**Figure 1H**). Of the leaf color mutants, we found a virescent leaf mutant that exhibited yellow leaf color at emergence and then gradually turned to green in approximately eight days(**Figure 1I**). We also screened a mutant with yellow true leaves throughout the growth period and compared the results with the WT, which grew normally (**Figure 1J**). We found two different types of sterile male mutants: one was a mutant with only vegetative branches and was unable to flower (**Figure 1D**), and the other was a mutant with fruiting branches that grew wildly without flowering and could not set bolls (**Figure 1E**). The inability to flower makes these mutants difficult to preserve.

In addition, three lethal mutants were screened that emerged normally but could not grow true leaves normally and died. Moreover, some mutations were found with variation in boll shape, boll size, and fiber length compared with the WT. Consequently, CIB radiation produced many types of mutants, some of which had positive traits that could be used as germplasm for cotton improvement.

### Genome-wide identification of DNA mutation

#### Identification of CIB-induced SBSs

To understand the genetic mutations induced by CIB, a WT line, Ji172, and its three mutagenized progeny with stable inheritance of traits (M_8_) were investigated using whole-genome sequencing. Three mutants were induced by 200 Gy CIB that had small leaf, yellow leaf, and semi-dwarf phenotypes. On average, 57 Gb were obtained for each sample (**Table S3**). The raw sequencing data for each sample was filtered and aligned with the TM-1 reference genome by BWA software. The average sequencing depth of each sample was more than 16-fold, and the similarity when mapped to reference genome was around 96%, with 1×, 5×, and 10× coverage ratios of around 82%, 80%, and 75%, respectively(**Table S3**).

**Figure 2:**
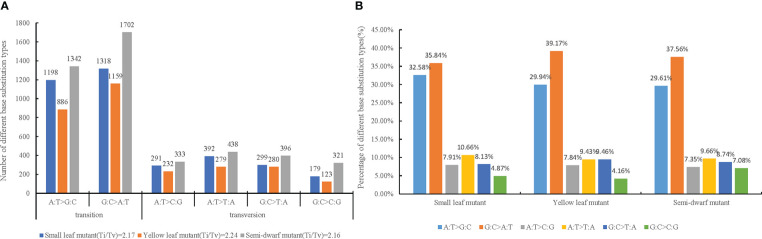
Transitions and transversions of induced by CIB irradiation among three mutation lines. A:T>G:C and G:C>A:T are transitions(Ti), A:T>C:G, A:T>T:A, G:C>T:A and G:C>C:G are transversions(Tv). **(A)** Number of different base substitution types and ratio of Ti/Tv in three mutation. **(B)** Percentage of different base substitution types in each mutation line.

After ruling out these background discrepancies, totals of 3677, 2959, and 4049 SBSs were identified from small leaf mutant, yellow leaf mutant, and semi-dwarf mutant, respectively (**Table S4**). To verify whether the mutations were indeed correct, five random mutation sites from semi-dwarf mutant including three SBS sites and two single base deletion/insertion sites were selected for Sanger sequencing. The results showed that those sites obtained by genome re-sequencing were real mutations (**Figure S1**). The substitutions were classified as transition (mutations among the same type of bases, such as purine to purine or pyrimidine to pyrimidine) or transversion (mutations among different types of bases, such as purine to pyrimidine or pyrimidine to purine). There were more transitions than transversions, and the ratios of transition to transversion (Ti/Tv) in the three mutants ranged from 2.16 to 2.24 (**Figure 2A**). The total number of A:T>G:C transitions was 3426, whereas that of G:C>A:T transitions was 4179. Therefore, there was slightly less A:T>G:C than G:C>A:T in this study. Among transversion types, the total number of A:T>C:G, A:T>T:A, G:C>T:A, and G:C>C:G were 856, 1109, 975, and 623, respectively. These data showed that there were substantially more A:T>T:A transversions compared with the other types, there were similar amounts of A:T>C:G and G:C>T:A transversions, and there were substantially fewer G:C>C:G transversions than the other three types (**Figure 2A**). Among the three mutants, the proportions of these six substitution types were very close, which indicated that the type ratio of SBSs caused by irradiation was relatively stable under the same dose and LET (**Figure 2B**).

### Characteristics of InDel mutations induced by CIB irradiation

**Figure 3:**
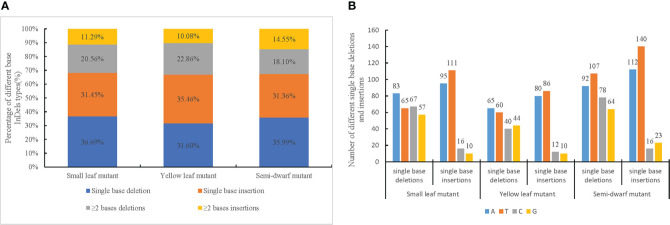
Ratio and type of InDels among three mutation lines. **(A)** Percentage of different base InDels types in each mutation line. **(B)** Number of different single base deletions and insertions in three mutants.

In addition to the SBS mutations, InDel mutations were also induced by CIB irradiation. A total of 756, 610, and 947 InDels were identified from the small leaf, yellow leaf, and semi-dwarf mutants, respectively (**Table S5**). In all three mutants, single-base deletions and insertions were the main types of InDel mutations. Of the InDels, 36.69% and 31.54% were single-base deletions and insertions, respectively, in the small leaf mutant; 31.60% and 35.46% were single-base deletions and insertions, respectively, in the yellow leaf mutant; and 35.99% and 31.36% were single-base deletions and insertions, respectively, in the semi-dwarf mutant (**Figure 3A**). Additionally, there were ≥2-base deletions and insertions. Of the identified InDels, 20.56% and 11.29% were ≥2-base deletions and insertions, respectively, in the small leaf mutant; 22.86% and 10.08% were ≥2-base deletions and insertions, respectively, in the yellow leaf mutant; and 18.10% and 14.55% were ≥2-base deletions and insertions, respectively, in the semi-dwarf mutant (**Figure 3A**).

The ratio of single-base InDels to ≥2-base InDels was more than 2-fold. Additionally, the number of single-base insertions and deletions was similar in each mutant, whereas there were nearly twice as many ≥2-base deletions than insertions. Finally, there were slightly more A and T single-base deletions in mutants than G and C single-base deletions, and there were substantially more A and T single-base insertions than G and C single-base insertions (**Figure 3B**); this indicated that A and T bases were more prone to insertion during mutagenesis.

### Distribution of mutations induced by CIB irradiation

#### Distribution of SBS mutations

The distribution of identified SBS mutations on cotton chromosomes ranged from one per 60.79 kb (A08) to 63,509.00 kb (D01) in the small leaf mutant, one per 78.51 kb (D04) to 42,865.22 kb (A04) in the yellow leaf mutant, and one per 52.60 kb (A04) to 48,997.75 kb (A07) in the semi-dwarf mutant (**Table S4**). There was no regularity in variant rate of SBSs across each chromosome in the three mutants. For example, on the A08 chromosome, there was one SBS variation per 60.79 kb in the small leaf mutant, while there was one SBS variation per 9,693.91 kb in the yellow leaf mutant and per 14,002.32 kb in the semi-dwarf mutant (**Table S4**).

**Figure 4:**
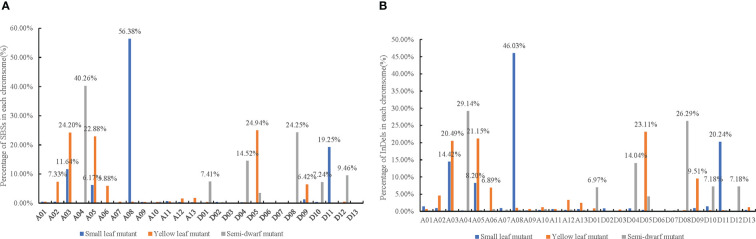
The pecentage of SBS and InDels mutations in each chromosome induced by CIB irradiation among three mutation lines. **(A)** The pecentage of SBS mutations in each chromosome among three mutation lines. **(B)** The pecentage of InDels mutations in each chromosome among three mutation lines.

**Figure 5:**
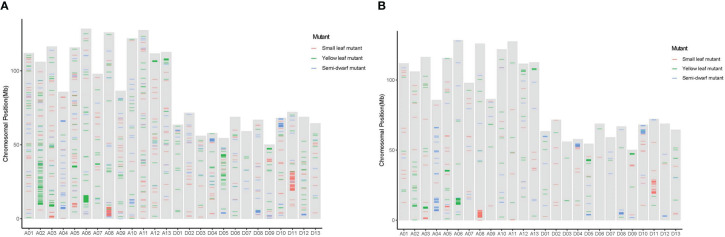
Distribution of SBS and InDels across all chromosomes mutations among three mutation lines. **(A)** The distribution of SBS mutations on each chromosome among three mutation lines. **(B)** The distribution of InDels mutations on each chromosome among three mutation lines.

The SBSs of each mutant covered all chromosomal segments. However, there were substantially more SBSs on some chromosomes than others. For example, the SBSs on chromosome A08 had 56.38% of all SBSs in the small leaf mutant; A03, A05, and D05 had 24.20%, 22.88%, and 24.94% of all SBSs, respectively, in the yellow leaf mutant; and A04 and D08 had more than 60% of all SBSs in the semi-dwarf mutant (**Figure 4A**). Moreover, there were “high-density” SBS regions at one or both ends of some chromosomes (**Figure 5A**). For example, the SBSs in a 19–20 Mb interval on chromosome A11 accounted for 18.8% of the chromosome in the small leaf mutant.

#### Distribution of InDel mutations

InDels were the other main mutation type induced by CIB. The distributions of identified InDel mutations on cotton chromosomes were similar to those of SBSs; they ranged from one per 362.13 kb (A08) to 68,832.47 kb (D12) in the small leaf mutant, one per 386.76 kb (D05) to 97,995.49 kb (A07) in the yellow leaf mutant, and one per 268.63 kb (D08) to 64,550.65 kb (D13) in the semi-dwarf mutant (**Table S5**).

The variant rates of InDels across each chromosome were also uneven. For example, A08 had 46.03% of InDels in the small leaf mutant; A03, A05, and D05 had 20.49%, 21.15%, and 23.11%, respectively, in the yellow leaf mutant; and A04 and D08 had 29.14% and 26.29%, respectively, in the semi-dwarf mutant (**Figure 4B**).

### Effect of variation on gene function

All of the verified SBS and InDels sites were annotated by SnpEff software. We found that most SBSs (approximately 59%-70%) occurred in intergenic regions. In addition, 13.28%-19.06% of mutations mapped to upstream gene regions, 13.28%-15.59% mapped to downstream gene regions, 1.59%-2.53% mapped to exons, and 1.86%-4.29% mapped to introns (**Table 2**). Furthermore, “high-density” InDels regions at one or both ends of some chromosomes (**Figure 5B**) and genome-wide annotation statistics of InDels indicated that approximately 48.50%-60.70% of InDels occurred in intergenic regions, 17.38%-23.47% occurred in upstream gene regions, 19.07%-20.90% occurred in downstream gene regions, 0.42%-0.81% occurred in exons, and 2.29%-5.81% occurred in introns (**Table 3**). The results showed that only low ratio of mutation occurred in missense, non-synonymous and stop-gain range, and these mutations involving functional gene regions may be the main variants that cause phenotypic mutations, and these results could be useful to explorer the candidate functional genes in further study.

**Table 2:** The locations on the genome of SBS mutations induced by CIB irradiation.

Type (alphabetical order)	Small leaf mutant		Semi-dwarf mutant		Yellow leaf mutant	
	Count	Percent	Count	Percent	Count	Percent
UPSTREAM	1,072	19.06%	535	13.28%	1,129	17.07%
DOWNSTREAM	875	15.56%	535	13.28%	1,031	15.59%
EXON	113	2.01%	64	1.59%	167	2.52%
INTERGENIC	3,315	58.94%	2,816	69.89%	4,057	61.35%
INTRON	241	4.29%	75	1.86%	224	3.39%
SPLICE SITE REGION	8	0.14%	4	0.10%	5	0.08%

**Table 3:** The locations on the genome of InDels mutations induced by CIB irradiation.

Type (alphabetical order)	Small leaf mutant		Yellow leaf mutant		Semi-dwarf mutant	
	Count	Percent	Count	Percent	Count	Percent
UPSTREAM	319	23.47%	167	17.38%	335	21.15%
DOWNSTREAM	284	20.90%	184	19.15%	302	19.07%
EXON	11	0.81%	4	0.42%	10	0.63%
INTERGENIC	660	48.57%	584	60.77%	866	54.67%
INTRON	79	5.81%	22	2.29%	68	4.29%
SPLICE SITE ACCEPTOR	1	0.07%	none	none	none	none
SPLICE SITE DONOR	1	0.07%	none	none	none	none
SPLICE SITE REGION	4	0.29%	none	none	3	0.19%

## Discussion

### CIB irradiation is useful for inducing cotton phenotype mutations

CIB irradiation has been widely used in many plants for crop breeding and research on molecular genetic mechanisms. However, heavy-ion radiation mutagenesis of cotton has not been reported. The mechanism of heavy-ion radiation mutagenesis is very complex, and the mutagenic effects are affected by many factors, such as ion dose, radiation energy and type, and the temporal pattern of exposure.

Kazama et al. (2011) reported that dry *Arabidopsis thaliana* seeds irradiated by carbon ions with LET of 30.0 KeV/μm at a dose of 400 Gy induced a higher mutation frequency than LET of 22.5 KeV/μm at 250 Gy and 450 Gy (Kazama et al., 2011). However, Yang et al. (2019) showed that LET of 50 KeV/μm at 80 Gy of ^12^C6^+^ ions was ideal for irradiating rice seeds to achieve LD50 (Yang et al., 2019). In chrysanthemum, a 320 MeV CIB with LET of 76 keV/µm was the most effective for inducing flower color mutants(Yamaguchi et al., 2010). In our study, to identify the most effective irradiation dose, we measured the survival rate for each of five radiation doses. The results showed that 200 Gy under 226.9 KeV/μm was the LD50 for cotton and induced much higher rates of variation. These results indicated that the optimal LET and radiation dose to induce mutations may vary among species.

We found many visible phenotypic mutants in treatments with radiation doses greater than 100 Gy in this study. Because we only recorded mutants that could be visually distinguished, the total number of mutations may be higher than the present data. For example, some mutants with altered genes may not have been identified, such as those mutants with altered stress resistance. In general, high-dose irradiation is known to negatively influence the traits of plants(Lee et al., 2021). Our results also showed that most mutants exhibited poor agronomic traits, such as the yellow leaf mutant, short fiber length mutant, and small boll size mutant. Although these phenotypes are not favorable, they may be useful for studying the mechanism of related agronomic traits.

### Genomic profiles of mutants induced by CIB irradiation

In this study, sequencing analysis was based on three mutagenized progeny lines (small leaf, yellow leaf, and semi-dwarf mutants) and a WT line. In fact, spontaneous mutation is truly existent, but the per-generation rates of spontaneous mutation genetic is under strong evolutionary constraint (Hofmeister et al., 2020). Previous studies showed that the spontaneous mutation rate of Arabidopsis thaliana is 7×10^−9^ base substitutions per site per generation, which is around 0.57 base substitution on average per generation (Ossowski et al., 2010). Whereas, the mutation rate of the 200 Gy CIB irradiation is significantly higher than spontaneous mutation in *Arabidopsis thaliana *(Du et al., 2017). In addition, it was reported that the per-year genetic mutation rates in *poplar* (1.99×10^-9^) is lower than in *Arabidopsis thaliana*, and the probable reason is that long-lived perennials may have evolved mechanisms to protect these cell types from the persistent influence of environmental mutagens (Du et al., 2017). Cotton is a perennial plant, so we speculated that the rate of spontaneous mutation of cotton may be between 1.99×10^-9^ and 7×10^-9^, the effect of which was negligible in the present study. To ensure the reliability of results using a Illumina HiSeqTM 2500 system, we focused on the SBSs and small InDels. Among the identified SBSs, the ratios of transitions to transversions were very similar and ranged from 2.16 to 2.24 (**Figure 2A**). This value was slightly lower than that reported for spontaneous mutations (2.41–2.73) (Ossowski et al., 2010), and close to that of CIB-induced mutations in rice (2.22) (Yang et al., 2019). In addition, these values were greater than those reported for rice mutations induced by γ ray (1.15–1.68) (Li et al., 2016), EMS mutagenesis (1.63–1.83) (Mohd-Yusoff et al., 2015), fast neutron induced mutations in *Arabidopsis* plants (0.86) (Belfield et al., 2012), CIB-induced mutations in *Arabidopsis* plants (0.99) (Du et al., 2017), and CIB irradiation-induced substitutions in *Saccharomyces cerevisiae* (0.746) (Guo et al., 2019). Consequently, there is considerable variation in ratios of transitions to transversions by inducing mutations using different techniques. However, regardless of the ion type and energy, the induced mutation ratios of transitions to transversions are always lower than the amount of spontaneous mutations. This indicates that artificial mutagenesis can balance the ratio of transitions and transversions.

In this study, the amounts of A:T>G:C and G:C>A:T transitions were similar, but differed from the reports of CIB-induced mutations and EMS mutagenesis in *Arabidopsis *(Henry et al., 2014; Du et al., 2017), for which the proportions of this kind of mutations were much higher. Among transversions, G:C>C:G occurred less than the other transversions, but the proportions of the other three types were similar to each other.

Among the other main mutation type, InDels, most mutations were single-base insertions and deletions, which had approximately 2-fold greater abundance than ≥2-base insertions and deletions. However, the proportions of single-base insertions and deletions were similar. Overall, the CIB-induced InDels accounted for 17.05%–18.96% of mutations, which was close to the GR-induced InDels in rice (17.85%) and lower than the CIB-induced InDels in rice (25.44%) (Yang et al., 2019). However, the mechanism underlying this discrepancy is still unclear, and more research is needed to resolve this issue.

We found that SBSs were the most abundant type of mutation induced by CIB. However, far fewer SBSs were induced by CIB compared with EMS. Lian et al. constructed a cotton EMS-treated mutant library and found more than 60,000 SNPs in an albino mutant (Lian et al., 2020). Similar mutational effects were found in *Arabidopsis* and rice (Martín et al., 2009; Serrat et al., 2014). Additionally, previous studies demonstrated that EMS induced more than 2,000 and 10,000 mutation sites in *Arabidopsis* and rice (Uchida et al., 2011; Sevanthi et al., 2018), whereas CIB only generated approximately 40 (Du et al., 2017)and 175 (Yang et al., 2019) substitutions per mutation in *Arabidopsis* and rice, respectively. These results indicate that CIB irradiation induces much fewer SNP mutations compared with EMS.

Despite the presence of fewer SBSs and InDels, we observed substantial phenotypic changes involving multiple morphological changes, such as leaf color, plant architecture, boll shape and size, fiber length, and fertility. Therefore, CIB may also cause types of mutations other than SBSs and InDels. It was reported that heavy-ion beams induce chromosomal rearrangement in high frequency rather than gene function loss, and most of the novel mutations produced by ion beam irradiation may not be caused by ordinary gene disruption but by chromosomal rearrangements (Kikuchi et al., 2009). However, second-generation high-throughput sequencing technology could not detect chromosomal rearrangements because of the short sequencing length. Therefore, further research needs to be carried out using third-generation sequencing technology.

### Mutation distribution on chromosomes

The distribution of ion radiation mutations on chromosomes is an important property and should be extensively analyzed (Li et al., 2016; Tan et al., 2019; Xiong et al., 2020). In this paper, the radiation-induced mutations were unevenly distributed across the cotton genome, and the number of variants was significantly higher on some chromosomes than others. For example, SBSs on chromosome A08 accounted for 56.38% of SBSs in the small leaf mutant; those on chromosomes A03, A05, and D05 accounted for 70% in the yellow leaf mutant; and those on A04 and D08 accounted for more than 60% in the semi-dwarf mutant. In addition, the distribution on chromosomes was also uneven; the number of SBSs in a 19–20 Mb interval on chromosome A11 accounted for 18.8% of the chromosome in the small leaf mutant, which was consistent with the findings of previous studies on mutations in wheat induced by LR (Li et al., 2016; Tan et al., 2019; Xiong et al., 2020) and mutations in rice induced by gamma rays and CIB (Yang et al., 2019).

The uneven distribution of mutations may be due to the properties of heavy-ion radiation. Heavy ions are high-LET-type particles that produce strong local ionization on their penetrating path. A previous study found that, after high-LET rays act on cells, a certain density of uneven energy deposition is generated in DNA molecules and forms several local DNA damage clusters, including apurinic/apyrimidinic sites, oxidized purines or pyrimidines, single-strand breaks, and double-strand breaks (O. Blaisdell et al., 2001; Wang et al., 2005; Wang et al., 2008; Brenner and Ward, 2009). Such localized multiple damage sites and small segments of fragmented DNA may be responsible for the uneven distribution of mutation sites.

## Conclusion

Mutation breeding and functional gene mapping are based on induction of genetic variations. CIB irradiation has been considered the most powerful source of mutagenesis. In this study, a set of strategies was established to create and identify cotton mutations caused by CIB irradiation. We explored the effect of different doses of high-intensity irradiation on inducing cotton mutations and constructed genomic mutation profiles induced by CIB irradiation. We will continue to develop mutation detection strategies for stably inherited traits that were selected upon mutation induction. We hope that our data provide valuable insights into a potential mechanism for plant mutation *via* CIB irradiation.

## Data availability statement

The data presented in the study are deposited in the SRA repository, accession number PRJNA887016.

## Author contributions

YW and HZ conceived and designed the project. JL and GZ designed the experiments and wrote the manuscript. JG performed the cotton mutagenesis. ZG, HD, XL, and ZA analyzed the data. All authors contributed to the article and approved the submitted version.

## Supplementary material

Supplementary Figure 1 Verification of mutations by Sanger sequencing. The sequence alignment of five random mutation sites including three SBS sites as well as two single base deletion/insertion sites from semi-dwarf mutant. **(A)** The SBS site at A01:79,923,043. **(B)** The SBS site at D09:11,692,693. **(C)** The SBS site at D12: 9,660,263. **(D)** The single base insertion at A03:30,466,682. **(E)** The single base deletion at D11:10,895,225
