# Supplementary figures and images for: Genome-wide analysis of mutations induced by carbon ion beam irradiation in cotton
Source: Front Plant Sci. 2023 Feb 16;14:1056662. doi: 10.3389/fpls.2023.1056662 (PMC9978701; doi:10.3389/fpls.2023.1056662)

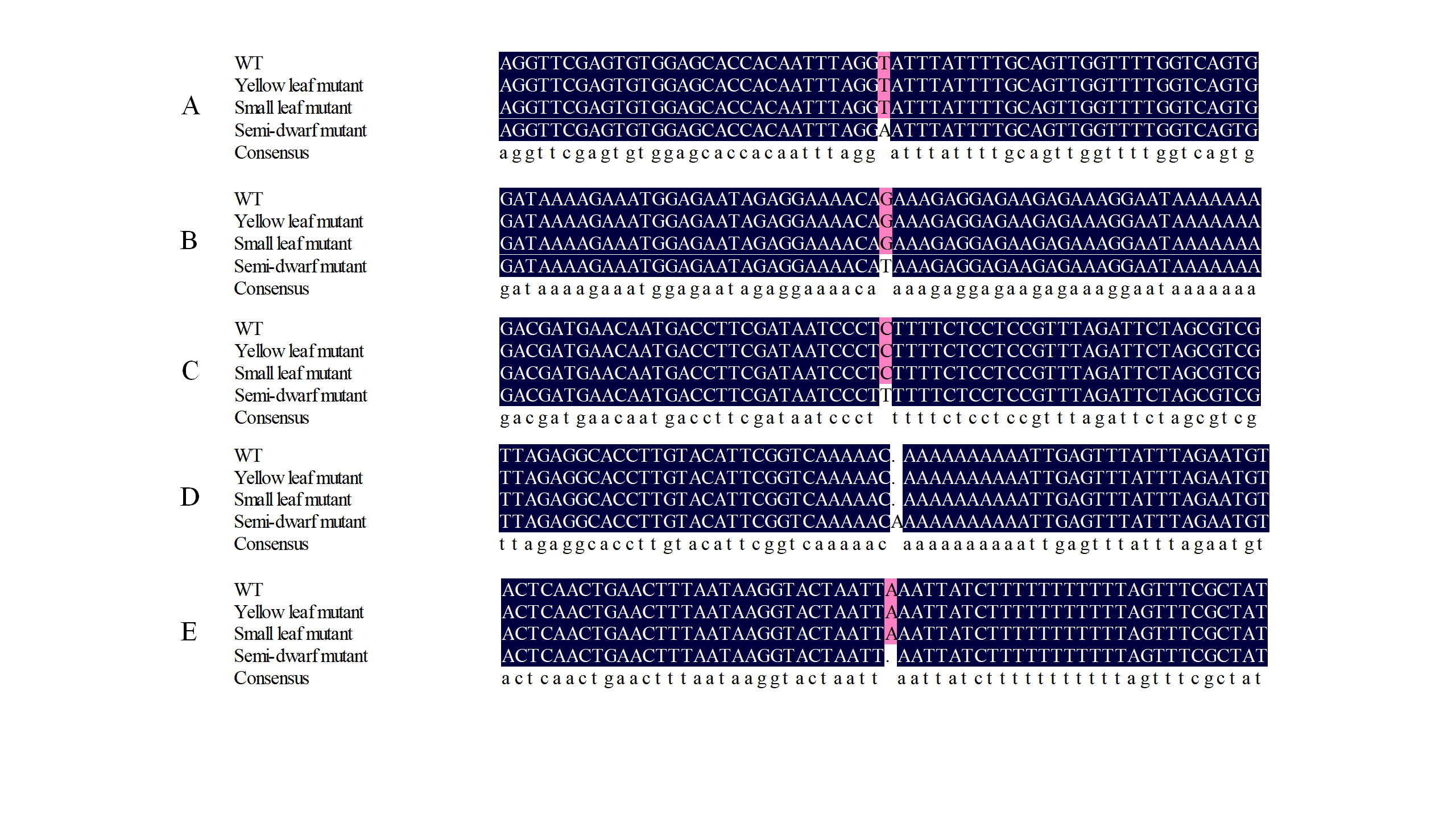

Supplement: Supplementary Figure 1 — Verification of mutations by Sanger sequencing. The sequence alignment of five random mutation sites including three SBS sites as well as two single base deletion/insertion sites from semi-dwarf mutant. (A) The SBS site at A01:79,923,043. (B) The SBS site at D09:11,692,693. (C) The SBS site at D12: 9,660,263. (D) The single base insertion at A03:30,466,682. (E) The single base deletion at D11:10,895,225 [file Image_1.tif]
